# Performance Evaluation of the MBT STAR^®^-Carba IVD Assay for the Detection of Carbapenemases With MALDI-TOF MS

**DOI:** 10.3389/fmicb.2019.01413

**Published:** 2019-06-20

**Authors:** Ahalieyah Anantharajah, Bastien Tossens, Nathalie Olive, Benoit Kabamba-Mukadi, Hector Rodriguez-Villalobos, Alexia Verroken

**Affiliations:** Department of Microbiology, Cliniques Universitaires Saint-Luc, Université catholique de Louvain, Brussels, Belgium

**Keywords:** carbapenemase detection, hydrolysis assay, MALDI-TOF MS, Gram-negative bacteria, performances, positive blood cultures

## Abstract

**Objectives:** The increasing rate of carbapenem resistance in Gram-negative bacteria is a major public health problem and rapid detection is essential for infection management. We evaluated the performances of the MBT STAR^®^-Carba IVD assay (Bruker Daltonics) to detect carbapenemase-producing organisms (CPO) from bacterial colonies and directly from positive blood culture bottles with MALDI-TOF MS.

**Methods:** We analyzed 130 strains with a reduced susceptibility to at least one carbapenem including 109 CPO (6 KPC, 27 NDM, 21 VIM, 1 IMP, 41 OXA-48-like, 8 OXA-23, 2 OXA-24/-40, and 2 OXA-58) and 21 non-CPO. The assay on colonies was performed with all 130 strains while the assay on spiked blood cultures was performed with 45 strains. Samples were prepared with the MBT STAR^®^-CARBA IVD kit and imipenem hydrolysis by the potential carbapenemase was analyzed with the MBT STAR^®^-BL module (Bruker Daltonics) on MALDI-TOF MS.

**Results:** Performed on colonies, the assay detected all carbapenemase-producing Enterobacteriaceae (*n* = 78), *Pseudomonas* spp. (*n* = 19) and *Acinetobacter* spp. (*n* = 12). All 21 tested non-CPO remained negative resulting in sensitivity and specificity of 100%. Performed on positive blood cultures, the assay detected all carbapenemase-producing Enterobacteriaceae (*n* = 23) and *Pseudomonas* spp. (*n* = 4) but missed 9/12 carbapenemase-producing *Acinetobacter* spp. However, a prolonged imipenem-incubation time of the strain pellet improved carbapenemase detection. Non-CPO from positive blood culture bottles remained negative (*n* = 5) with the assay with the exception of one *Klebsiella pneumoniae* isolate.

**Conclusion:** The MBT STAR^®^-Carba IVD assay is a highly reliable method for the detection of carbapenemase activity in Gram-negative bacteria. However, time-consuming sample preparation steps and reagent costs need to be considered before implementation in a routine clinical microbiology laboratory.

## Introduction

Carbapenemase-producing organisms (CPO) are a worldwide threat for clinical patient care and public health. The mobile genetic elements bearing the carbapenemase genes are not only responsible for their rapid dissemination but also carry non-β-lactam resistance determinants hereby giving rise to extremely drug resistant isolates (Kumarasamy et al., 2010; Thomson, 2017).

Rapid and reliable detection methods allow appropriate antimicrobial therapy and early implementation of infection control measures to prevent subsequent dissemination of CPO. However, detection of carbapenemases has become a critical challenge in clinical microbiology. Nearly 20% of carbapenemase-producing isolates can be missed using interpretative criteria recommended by the EUCAST or the Clinical and Laboratory Standards Institute (CLSI) (Huang et al., 2014). Most carbapenemases hydrolyze the different carbapenems at variable levels hereby not systematically conferring full resistance. Besides, decreased carbapenem susceptibility may also be caused by reduced permeability due to porin down-regulation or over-expression of efflux pumps (Nordmann et al., 2012). Numerous phenotypic and DNA-based methods have been used in the laboratory aiming at the detection of CPO (Hrabak et al., 2014). Classically, results of culture-based phenotypic methods are available in 24–72 h after isolation of the bacteria from the infected samples, whereas PCR assay results are available within hours but at higher cost (Queenan and Bush, 2007). Moreover, PCR can only detect a predefined range of carbapenem-resistance genes and their presence does not guarantee their expression (Pellegrino et al., 2008). Recently rapid phenotypic methods have been developed such as hydrolysis methods allowing the recognition of all types of carbapenemases with optimal sensitivity and specificity (Dortet et al., 2014a; Tamma and Simner, 2018). These tests include biochemical tests and matrix-assisted laser desorption ionization time-of-flight mass spectrometry (MALDI-TOF MS) techniques. Following a short incubation of the Gram-negative strain with a defined carbapenem, MALDI-TOF MS monitors the distinct mass peaks of the hydrolysed and non-hydrolysed forms of the antibiotic in the bacterial suspension (Carvalhaes et al., 2013, 2014). Several arduous in-house hydrolysis MALDI-TOF MS assays have been described with different sets of antibiotic combinations, buffers, and variable incubation times from 15 min to 4 h (Hrabak et al., 2011; Mirande et al., 2015).

In the present study, we evaluated the commercial MBT STAR^®^-Carba IVD assay (Bruker Daltonik GmbH, Bremen, Germany) for MALDI-TOF MS carbapenemase detection. Testing was performed on culture isolates and directly on positive blood culture bottles.

## Materials and Methods

### Strain Collection

A total of 130 Gram-negative isolates, including 89 Enterobacteriaceae (Supplementary Table S1), 29 *Pseudomonas* spp. and 12 *Acinetobacter baumannii* complex, intermediate or resistant to at least one carbapenem (imipenem, meropenem or ertapenem) were selected to evaluate the performances of the MBT STAR^®^-Carba assay on colonies (Table 1A) These strains included all carbapenem resistant isolates recovered from clinical and screening samples at the microbiology laboratory of the Cliniques universitaires Saint-Luc between January 2015 and March 2018. Duplicate isolates from the same patient and duplicate outbreak strains were excluded. They were characterized for antimicrobial susceptibility with the automated Phoenix system (Becton-Dickinson, Franklin Lakes, NJ, United States) and confirmed with the manual disk diffusion method (Bio-Rad, Marnes-la-Coquette, France) using the EUCAST 2018 clinical breakpoints. Ultimately a PCR, as described below, used as reference method in this study, was performed on each strain to detect the presence of carbapenemase resistance genes. Included isolates carried a carbapenemase resistance gene (*n* = 109) or expressed other resistance mechanisms (*n* = 21) mainly including extended-spectrum-β-lactamases (ESBL) (*n* = 7), AmpC-type cephalosporinases (*n* = 3) associated or not with uncharacterized carbapenem resistance mechanisms (porin loss, efflux pumps overexpression) (*n* = 11). For the evaluation of the MBT STAR^®^-Carba assay directly on positive blood cultures, 45 isolates were selected among this collection (Table 1B).

**Table 1:** Performances of the MBT STAR^®^-Carba IVD Assay for the detection of carbapenem hydrolysis in carbapenem resistant organisms.

	Carbapenemase	No. of isolates	Incubation time (min)	Assay results			
				Hydrolysed	Non-hydrolysed	Sensitivity (95% CI*)	Specificity (95% CI*)
**A. Assay performed on bacterial colonies**							
**Enterobacteriaceae (*n* = 89)**						100% (95.3–100.0)	100% (74.1–100.0)

*K. pneumoniae* (*n* = 49), *E. coli*	OXA-48-like	41	30	41	–		
(*n* = 22), *K. oxytoca* (*n = 5*)	NDM	27	30	27	–		
*E. cloacae* (*n* = 6), *C. freundii*	KPC	6	30	6	–		
(*n* = 4), *E. aerogenes* (*n* = 2),	VIM	4	30	4	–		
*P. stuartii* (*n* = 1)	Negative	11	30	–	11		

***Pseudomonas* spp. (*n* = 29)**						100% (82.4–100.0)	100% (74.1–100.0)

*P. aeruginosa* (*n* = 28),	VIM	17	30	17	–		
*P. chlororaphis* (*n* = 1)	GES (ESBL)	1	30	–	1		
	IMP	1	30	1	–		
	Negative	10	30	–	10		

***A. baumannii* complex (*n* = 12)**						100% (75.7–100.0)	NA^#^

	OXA-23	8	60	8	–		
	OXA-24/-40	2	60	2	–		
	OXA-58	2	60	2	–		

**B. Assay performed on positive blood cultures**							
**Enterobacteriaceae (*n* = 26)**						100% (85.7–100.0)	66.7% (20.8–93.8)

*K. pneumoniae* (*n* = 16),	OXA-48-like	12	60	12	–		
*E. coli* (*n* = 5), *E. cloacae*	NDM	5	60	5	–		
(*n* = 2), *C. freundii* (*n* = 2),	KPC	4	60	4	–		
*E. aerogenes* (*n* = 1)	VIM	2	60	2	–		
	Negative	3	60	1	2		

***Pseudomonas* spp. (*n* = 7)**						100% (51.0–100.0)	100% (43.8–100.0)

*P. aeruginosa* (*n* = 6),	VIM	3	60	3	–		
*P. chlororaphis* (*n* = 1)	IMP	1	60	1	–		
	Negative	3	60	–	3		

***A. baumannii* complex (*n* = 12)**						25%/58.3% (8.3–53.2)/(31.9–80.7)	NA^#^

	OXA-23	8	60/120	2/4	6/4		
	OXA-24/-40	2	60/120	–/2	2/–		
	OXA-58	2	60/120	1/1	1/1		

*95% CI: 95% confidence interval. ^#^NA: non-applicable.

### Detection of Genes Encoding Carbapenemases

The carbapenemase resistance genes (*bla*_OXA-48_, *bla*_KPC_*, bla*_NDM_*, bla*_V IM_*, bla*_NDM_, and *bla*_IMP_) were detected by an in-house multiplex PCR (Bogaerts et al., 2013). To differentiate between the OXA-48 variants, the *bla*_OXA-48_-like genes were sequenced using the following primers: OXA-48F, 5′-ATGCGTGTATTAGCCTTATCG-3′ and OXA-48R, 5′-GAGCACTTCTTTTGTGATGGC-3′ (Bogaerts et al., 2013; Oueslati et al., 2015). The full sequence length was 774 bp. For carbapenemase-producing *A. baumannii* complex strains, detection of carbapenemase genes (*bla*_OXA-23_*, bla*_OXA-24_, and *bla*_OXA-58_) was performed by the Belgian national reference center.

### Blood Culture Preparation

Blood cultures were spiked by inoculating the bottles [Bactec Plus Aerobic, Lytic Anaerobic or Peds Plus media (Becton Dickinson)] with 10 mL of human blood and 20 μL of a 0.5 McFarland suspension from a fresh overnight culture isolate. Blood culture bottles were incubated directly in a Bactec FX blood culture system (Becton Dickinson) until they flagged positive. Bacterial material was isolated through an adapted Sepsityper (Bruker Daltonik GmbH) workflow: the amount of Lysis Buffer was reduced and no ethanol precipitation was performed to conserve the carbapenemase activity. Briefly, 1 mL of blood culture fluid was mixed with 100 μL Lysis Buffer. After vortexing, the mix was centrifuged at 14,000 rpm for 2 min, the supernatant was discarded, and the pellet was washed with 1 mL Washing Buffer. The extracted bacterial pellets were used to perform MALDI-TOF MS identification and MBT STAR^®^-Carba assay.

### MBT STAR^®^-Carba IVD Kit

**FIGURE 1:**
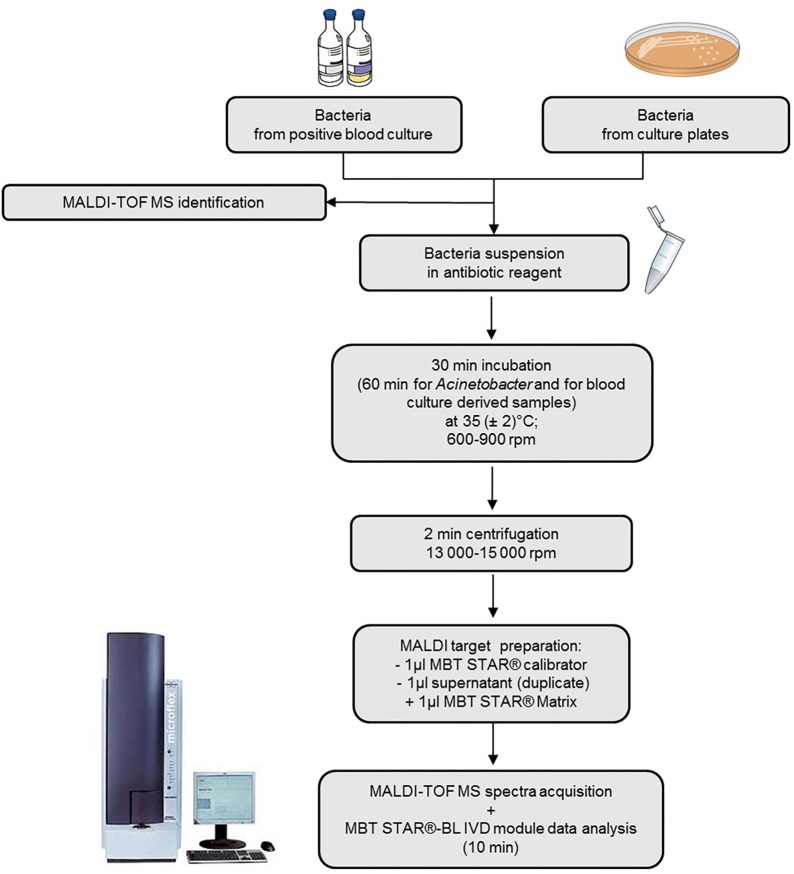
Scheme illustrating the MBT STAR^®^-Carba IVD workflow from colonies and from spiked blood cultures. One to five colonies from overnight cultures or the pellet obtained from positive blood cultures by the MBT Sepsityper kit were mixed with the reconstituted MBT STAR^®^-Carba Antibiotic Reagent containing imipenem. After incubation and centrifugation, cell-free supernatant is spotted onto a MALDI target plate and overlaid with matrix. Spectra are then acquired using the MALDI Biotyper^®^smart system and analyzed by the MBT STAR^®^-BL IVD module.

**FIGURE 2:**
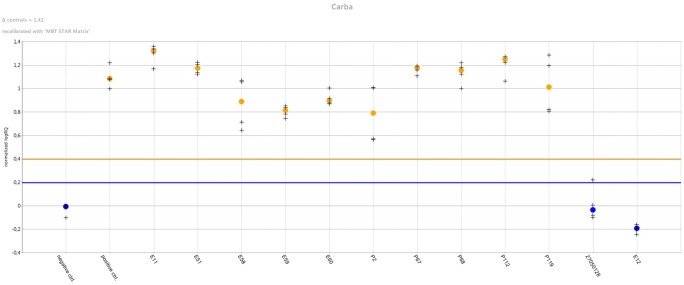
Results overview in the MBT STAR^®^-BL Software Module. The software monitors the carbapenemase activity on acquired mass spectra (4 mass spectra per isolate) by automatic calculation of the intact Imipenem intensity and corresponding ratio hydrolysed / non-hydrolysed. Results are normalized to signal intensities obtained from positive and negative control strains (normalized logRQ value). Color-coded plot indicates presence (orange) or absence (blue) of carbapenemase activity in bacterial strains with the horizontal lines representing the limit for positive and negative results.

The MBT STAR^®^-Carba IVD kit (Bruker Daltonik) was tested according to the manufacturer’s instructions. Briefly one to five colonies from overnight cultures or the pellet obtained from positive blood cultures was mixed with the reconstituted MBT STAR^®^-Carba Antibiotic Reagent containing imipenem (0.25 mg/mL). After incubation at 35°C under agitation for 30 min (60 min for *Acinetobacter* spp. and for blood culture derived samples), the reaction mixture was centrifuged and 1 μL of the supernatant was spotted in duplicate onto the MALDI target. Dried spots were overlaid with MBT STAR^®^Matrix and were analyszed on the MALDI Biotyper smart system (Bruker Daltonik GmbH) with the MBT STAR^®^-BL IVD module (Figure 1). This software provides an interpretation of the carbapenemase activity of each tested isolate based on the imipenem hydrolysis intensity compared with a negative and positive control strain, respectively, *Escherichia coli* ATCC 25922 and a characterized clinical KPC-producing Klebsiella pneumoniae (Figure 2). Discordant results between the assay and the molecular resistance gene identification led to repetition of both methods.

### Ethics Statement

Testing was performed in accordance with the ethical standards of the Cliniques universitaires Saint-Luc, in accordance with the ethical standards of the national research committee and in accordance with the 1964 Helsinki declaration and its later amendments. Information from microbiological and clinical files was anonymously analyzed and did not require patient’s informed consent.

## Results

All MBT STAR^®^-Carba Assay results are detailed in Table 1. Evaluated on bacterial colonies (Table 1A) the assay showed 100% of specificity and sensitivity for the detection of carbapenemase activity in Enterobacteriaceae. In particular, all OXA-48-like producers including 37 OXA-48, 3 OXA-181 and one OXA-244 were correctly detected. No carbapenemase activity was detected among isolates being resistant to carbapenems but non-carbapenemase producers. Our evaluation of the assay on 41 non-fermentative Gram-negative bacilli also allowed optimal sensitivity and specificity results of both 100%. However, the absence in our study of non-carbapenemase-producing *A. baumannii* complex strains yet resistant to carbapenems, didn’t allow the evaluation of the test’s specificity for this group.

From positive blood cultures (Table 1B), the MBT STAR^®^-Carba Assay yielded positive results for the isolates harboring KPC (*n* = 4), metallo-β-lactamase (*n* = 11) or OXA-48-like (*n* = 12) genes. All carbapenemase-producing Enterobacteriaceae (*n* = 23) were successfully detected with the assay. One repeated false-positive result among the three non-carbapenemase Enterobacteriaceae was observed with a *K. pneumoniae* isolate expressing a SHV ESBL, conferring resistance to ertapenem but still susceptible to imipenem and meropenem. Regarding non-fermenters, the assay correctly assigned as carbapenemase-producers all blood cultures growing with *Pseudomonas* spp. (*n* = 4*)* but only 25% (3/12) of bottles containing *A. baumannii* complex isolates while all of them yielded positive results when tested from bacterial colonies. Extending imipenem incubation time to 2 h increased sensitivity to 58.3% (7/12) in this complex. Four OXA-23 and one OXA-58 producers remained negative. Non-specific imipenem hydrolysis related to prolonged incubation times was excluded by obtaining negative results with five wild-type *A. baumannii*.

## Discussion

There is an urgent need for rapid and accurate detection of CPO. The selection of a carbapenemase detection test is dependent on several factors, including local carbapenemase prevalence, organisms to be tested (i.e., Enterobacteriaceae and/or non-fermenters), labor intensity, necessary equipment, reagent preparation requirements, cost, performances, and turnaround time (TAT) of the test (Tamma and Simner, 2018). The TAT is especially important to prevent unnecessary treatments and rapid spread of CPO in hospital settings.

The MBT STAR^®^-Carba assay evaluated in our study demonstrated excellent performances for Enterobacteriaceae, *Pseudomonas* spp. and *A.* *baumannii* complex isolates from bacterial colonies. All tested carbapenemases were correctly detected and a negative result correctly excluded the presence of carbapenemase ultimately preventing further unnecessary testing on such isolates. Similarly, two recent studies reported 100% sensitivity and a specificity between 98.2 and 100% after evaluation of the assay on Enterobacteriaceae isolates from solid media (Dortet et al., 2018; Rapp et al., 2018). Rapp et al. further reported that the MBT STAR^®^-Carba correctly assigned 4/4 carbapenemase-positive *P. aeruginosa* tested isolates but only 4/8 carbapenemase-positive *A. baumannii* tested isolates. This assay’s performances from bacterial colonies were similar to the performances of in-house developed MALDI-based methods particularly those developed with NH_4_CO_3_ increasing the catalytic efficiency of the OXA-48-like enzymes and therefore, their detection (Kempf et al., 2012; Papagiannitsis et al., 2015; Dortet et al., 2018; Miltgen et al., 2018; Oviano and Bou, 2019). OXA-48-like enzymes, known to possess a weak carbapenemase activity, are important to detect taking into account the large dissemination of OXA-48-like producers in Europe (Albiger et al., 2015). Moreover, the MBT STAR^®^-Carba assay showed superior sensitivity values than commercial colorimetric techniques for the detection of carbapenemases in Enterobacteriaceae and *A. baumannii* complex strains (ranging from 73 to 100% and from 21.0 to 86.0%, respectively). False negative results with these colorimetric assays occurred primarily with class D enzymes (Dortet et al., 2015; Noel et al., 2017; Simner et al., 2017; Tamma and Simner, 2018).

We additionally evaluated the applicability of the MBT STAR^®^-Carba assay performed directly on spiked blood cultures. Delays in appropriate antimicrobial treatment contribute to increased mortality of septic patients. Compared to phenotypic carbapenemase-detection approaches requiring cultured material, the assay performed directly on the blood bottles flagged positive drastically reduced time to results with more than 24 h. As observed from bacterial colonies, the assay detected 100% of the tested carbapenemase-producing Enterobacteriaceae and *Pseudomonas* spp. Our results stand out in comparison with colorimetric approaches reporting sensitivities between 64.0 and 91.3% for OXA-48-like CPO detection directly from positive blood cultures (Dortet et al., 2014b; Lima-Morales et al., 2018). One *K. pneumoniae* with a positive MALDI-TOF MS-based test and a negative PCR assay was observed in this study. However, precautions need to be taken when using the assay for the detection of carbapenemase-producing *A. baumannii* complex directly from positive blood cultures as the sensitivity after a 2 h incubation is only 58.3%. These results were in line with previous studies of in-house MALDI-TOF MS assays that demonstrated poor performances ranging from 27 to 63.2% for the detection of carbapenemase producing *A. baumannii* complex directly from positive blood cultures (Carvalhaes et al., 2014; Ghebremedhin et al., 2016). This could be explained by poor efficiency of Class D carbapenem-hydrolyzing β-lactamase (CHDL) (OXA-23, OXA-24/-40, and OXA-58) to hydrolyze the β-lactam ring of carbapenem antibiotics (Potron et al., 2015). Approaches to improve the assay for example increasing the bacterial inoculum size, increasing incubation time and modifying the Sepsityper bacterial extraction process might be valuable.

MALDI-TOF MS has become a well-established rapid identification tool and the simultaneous use of the system for carbapenemase detection seems convenient. It has the potential to detect carbapenemase activity regardless of the produced enzyme, including novel enzymes. Compared to the diversity of previous MALDI-TOF MS based in-house protocols (Mirande et al., 2015), the MBT STAR^®^-Carba IVD assay provides a fast and standardized method. Although the cost of the MBT STAR^®^-BL module needs to be considered, the automated interpretation with the software could be helpful for non-experienced users facing difficulties with spectrum analysis. The assay also overcomes the subjective visual interpretation of colorimetric assays, particularly in case of weak positive reactions leading to lower sensitivity for detection of OXA enzymes or more uncommon carbapenemases. The main drawback of the MBT STAR^®^-Carba assay is the hands-on time required for reagents and sample preparation during the pre-analytical procedure. Due to the relative instability of the imipenem solution, the MBT STAR^®^-Carba Antibiotic Reagent requires a reconstitution step before each use (Choquet et al., 2018; Oviano and Bou, 2019). Additionally, the laboratory has to possess a fresh culture of a carbapenemase-producing strain as positive control to perform the test.

The MALDI-TOF MS assay exclusively detects enzymatic carbapenem resistance but does not detect carbapenem resistance due to other mechanisms (i.e., efflux pump, porin loss) and extended phenotypic susceptibility testing is still required to define the antibiotic treatment options for the infected patient. Likewise, additional techniques are required for precise carbapenemase gene identification. The characterization of the carbapenemase type is of growing interest not only for epidemiology purposes but also for antimicrobial decisions. Indeed, some new β-lactamase inhibitor combinations in the pipeline as ceftazidime-avibactam or meropenem-vaborbactam are active on class A (KPC) and class D (OXA-48-like) carbapenemases but have no efficacy on metallo-β-lactamases (Liscio et al., 2015). Recent publications demonstrated the potential use of the MALDI-TOF MS platform for carbapenemase classification (Oviano et al., 2016; Cordovana et al., 2018) or for rapid AST independently of underlying resistance mechanisms (Idelevich et al., 2018). We would like to expect the development of a combined MALDI-TOF MS process allowing consecutive bacterial identification, AST, resistance detection, and classification of beta-lactamases.

The isolate collection tested in this study was a reflection of our local CPO epidemiology and was limited in the diversity of carbapenemase types. Therefore, additional testing of isolates carrying less common carbapenemase genes (*bla_OXA-51_* with an upstream insertion of IS*Aba, bla_GES_*, *bla_IMI_, bla_NMC_*, and *bla_SPM_*) is warranted to confirm our analytical observations. In additional, a prospective evaluation of the MBT STAR^®^-Carba assay on consecutive clinical samples should be performed in future studies.

In conclusion, our results showed that the MBT STAR^®^-Carba IVD assay is a reliable approach for the detection of carbapenemase-producing strains and may provide an answer within hours for antimicrobial therapy adjustment and early implementation of infection control measure.

## Author Contributions

AV and AA contributed to the conception and design of the study. AA, BT, and NO performed the experiments. AA and AV analyzed all the experiments. BK-M contributed to the design and analysis of DNA sequencing. AA wrote the manuscript. HR-V and AV provided critical feedback. AV contributed to the final version of the manuscript.

## Conflict of Interest Statement

The authors declare that the research was conducted in the absence of any commercial or financial relationships that could be construed as a potential conflict of interest.
