# Supplementary material for: Performance Evaluation of the MBT STAR®-Carba IVD Assay for the Detection of Carbapenemases With MALDI-TOF MS
Source: Front Microbiol. 2019 Jun 20;10:1413. doi: 10.3389/fmicb.2019.01413 (PMC6596351; doi:10.3389/fmicb.2019.01413)
Supplement: Supplementary file 1 [file Table_1.docx]

| **Table S1.** Distribution of carbapenemase and non-carbapenemase-producing Enterobacteriaceae isolates (n=89). CPE: Carbapenemase-producing Enterobacteriaceae | | | | | | | | |
| --- | --- | --- | --- | --- | --- | --- | --- | --- |
| **Enterobacteriaceae** | **Total number of isolates** | **CPE** | | | | | | **Non**  **CPE** |
|  |  | **OXA-48-like** | | | **KPC** | **NDM** | **VIM** |  |
|  |  | OXA-48 | OXA-181 | OXA-244 |  |  |  |  |
| *Klebsiella pneumoniae* | 49 | 21 | 1 |  | 5 | 18 | 1 | 3 |
| *Escherichia coli* | 22 | 9 | 2 | 1 |  | 5 | 1 | 4 |
| *Klebsiella oxytoca* | 5 | 2 |  |  | 1 |  |  | 2 |
| *Enterobacter cloacae* | 6 | 4 |  |  |  |  | 1 | 1 |
| *Citrobacter freundii* | 4 | 1 |  |  |  | 2 | 1 |  |
| *Enterobacter aerogenes* | 2 |  |  |  |  | 1 |  | 1 |
| *Providencia stuartii* | 1 |  |  |  |  | 1 |  |  |
